# Assessment of potential for viral contamination of user and environment *via* aerosols generated during hand drying: A pilot study

**DOI:** 10.3389/fpubh.2022.1010802

**Published:** 2022-10-25

**Authors:** Ines B. Moura, Karen Bentley, Mark H. Wilcox

**Affiliations:** ^1^Faculty of Medicine and Health, Leeds Institute of Medical Research, University of Leeds, Leeds, United Kingdom; ^2^Department of Microbiology, Leeds Teaching Hospitals NHS Trust, Leeds General Infirmary, Leeds, United Kingdom

**Keywords:** virus transmission, viral contamination, hand drying, paper towels, jet air dryer

## Abstract

**Background:**

Hand drying is an essential step of hand hygiene, helping remove microbes remaining on hands following handwashing. However, it is unclear whether particles dispersed or aerosolized during hand drying can also have an impact on microbe dissemination and so pose an infection risk.

**Methods:**

We used a PR772 bacteriophage to investigate whether microorganisms remaining on hands can disperse in the washroom environment and contaminate facemasks of others sharing the same space, as a surrogate for virus inhalation risk. Hand drying using either a jet air dryer or paper towels were performed, and mask contamination by splattering and droplet deposition was investigated, up to 15 min following each procedure.

**Results:**

Facemask contamination by splattering was 10-fold higher when a jet air dryer was used, compared with hand drying by paper towels, for both the person performing the hand drying and for standby users stationed at 1 and 2 m distance. Facemask contamination by droplet/aerosols deposition was higher in the first 5 min following hand drying, for both methods; however, virus load was significantly higher when a jet air dryer was used. In the jet air dryer assays, facemask contamination increased at 15 min post-hand drying, suggesting aerosolization of small particles that remain airborne for longer.

**Conclusion:**

When using a jet air dryer, virus contamination dispersed further and for a longer period of time (up to 15 min post hand-drying). The method chosen for hand drying can potentially impact the airborne dissemination of microbial pathogens, including respiratory virus, and so potentially increase the risk of exposure and infection for other washroom users.

## Introduction

The coronavirus disease (COVID-19) increased awareness to the importance of hand hygiene in reducing dispersion of microbes in public spaces. Previous studies showed that jet air dryers have a higher potential to contaminate the standby user than other hand drying methods, such as paper towels (1, 2). This is consistent with droplet splattering close to the hand dryers and wider dispersal of smaller particles (aerosols) during hand drying (3). When performing air sampling of the washroom environment, it was found that aerosolized bacteria dispersed from the jet air dryer unit to a distance up to 1 m, within the first 5 min of air sampling. Furthermore, bacteria persisted in the air beyond the hand drying time, with approximately half of the indicator bacteria used in the study (lactobacilli) being collected beyond 5 min after drying had completed (1).

Surgical masks and N95 (FFP2) masks remain widely used in healthcare settings to aid protection of staff and patients against infection by SARS-CoV-2 (4–6). A simulation study examining mask efficiency when infectious SARS-CoV-2 was exhaled as droplets/aerosols found that N95 have the highest protective effect (7). Higher efficacy of N95 masks compared with surgical masks has previously been reported also in COVID-19 positive patients (8). The use of N95 masks increased with the dispersion of the SARS-CoV-2 Omicron variant (5) following recommendations by the Centers for Disease Control and Prevention and the World Health Organization (9, 10).

Given the potential of jet air dryers to disperse large and small droplets/aerosols to the washroom environment during hand drying (1, 3), it is possible that viral particles will deposit on masks of other public toilet users, thus promoting virus dissemination. However, this research requires a model microorganism to simulate virus in the air and washroom environment.

Bacteriophage PR772 is innocuous to humans and the environment (11, 12) and has been successfully used to measure microorganism survival on hands and surfaces, and environmental contamination in healthcare settings (2, 13). It reportedly has a capsid size of around 63–82 nm depending on measurement type, being smaller than retroviruses, influenza and other respiratory virus such as SARS-CoV-2 (80–100 nm) (12, 14, 15), so its size means it represents an extreme condition for a filtering material, such as facemask (respirator). In this pilot study, we propose to investigate if PR772 particles dispersed in the air can deposit and be recovered from the surface of facemasks, i.e., a non-porous surface acting as filter, acting as a surrogate for potential inhalation of virus.

We utilized a PR772 bacteriophage as an indicator to investigate whether microorganisms still present on hands following poor hand wash, can disperse in the washroom environment during hand drying using either a jet air dryer or paper towels, and contaminate facemasks of other users sharing the same environment.

## Methods

### Preparation of bacteriophage filtrate

Bacteriophage PR772 (BAA-769-B1) and host strain *Escherichia coli* K12 (BAA-769) were prepared as previously described (2). Bacteriophage filtrate was diluted to 10^7^ pfu/ml and kept at 4°C until use.

### Bacteriophage dispersion and aerosol formation during hand drying

Hand drying tests were carried out in a windowless room measuring 65 m^3^ and located at the Leeds General Infirmary (U.K.) (1). The room is rectangular-shaped with a paper towel dispensing unit and a hand dryer unit located on each side of the sink (Supplementary Figure 1), all located on the opposite side of the room entrance door. Room air was maintained by natural ventilation, without positive or negative pressure or air-conditioning; room average temperature during assays was 23.1°C with a relative humidity of 43%. Before each experiment, all surfaces were sanitized with chlorine wipes (Medipal, Pal, U.K.). For each test, participants' gloved hands were sanitized with 70% alcohol hand gel (Purell, U.K.) before immersion in a 10^7^ pfu/mL PR772 filtrate solution to represent poorly or unwashed hands. Hand drying was performed using either 4 paper towels (Hand Towels H3, Tork, U.K.), or a jet air dryer for 10s (Airblade^TM^ AB01, Dyson, U.K.). The jet air dryer used in the experiments was a blade hand dryer unit without ultraviolet light, providing hand drying through high speed, unheated air (Supplementary Figure 1).

**Figure 1:**
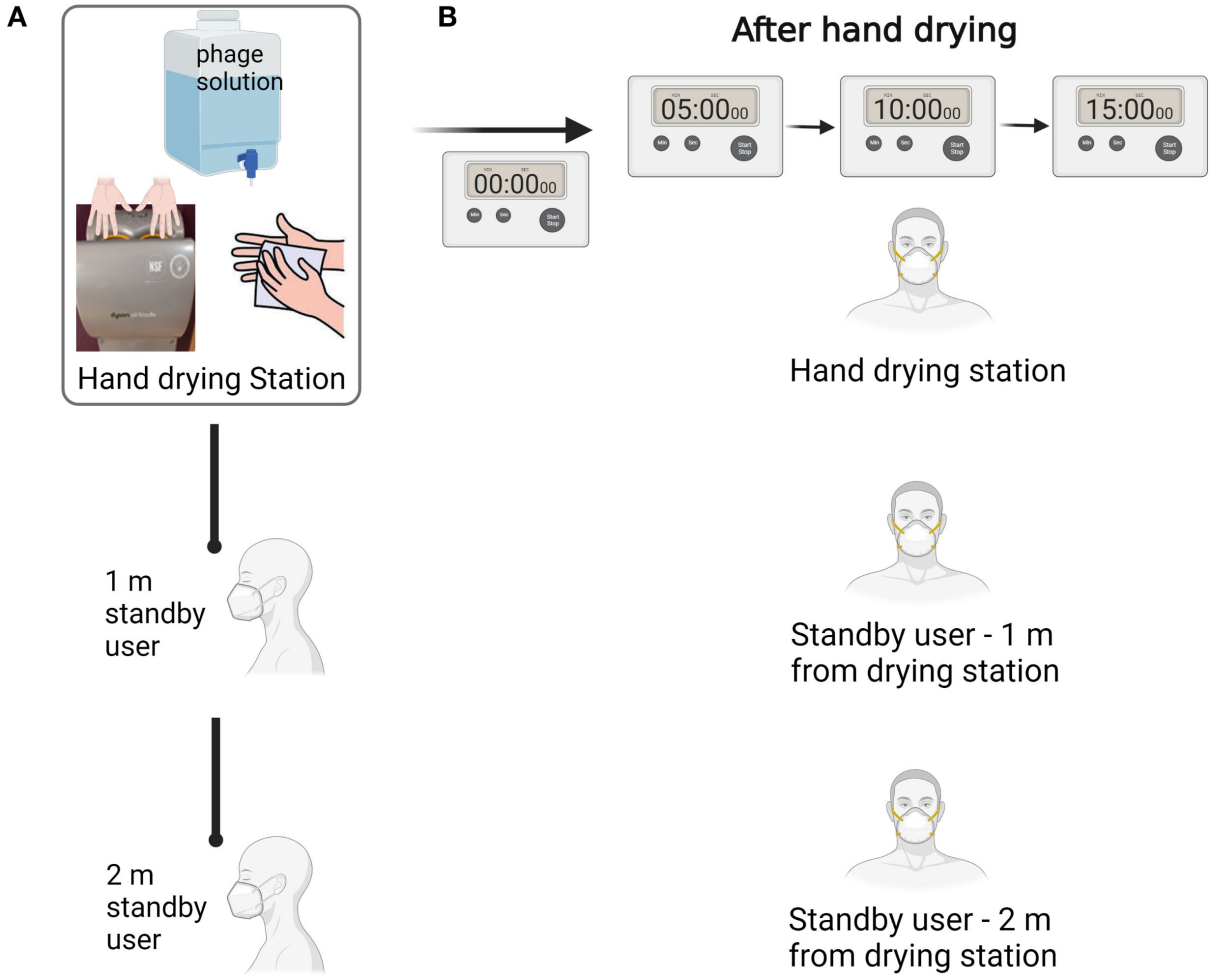
Schematics of the hand drying test. **(A)** Hand drying assay procedure to assess bacteriophage dispersion to N95 masks. **(B)** Experimental procedure to assess droplets/aerosol deposition on masks in three successive 5 min intervals. Figure created with Biorender.com.

Overall, 24 hand drying assays were performed by a total of 14 volunteers. Assays were spaced between January 2022 and May 2022; 12 assays were performed with paper towels and 12 assays were performed using jet air dryer. During hand drying, the room was shared by two additional volunteers acting as standby users and stationed at 1 and 2 m from the hand drying point (Figure 1A). All 3 participants in each assay wore a N95 respirator (F621, JSP, UK) during hand drying. Once hand drying was completed, respirators of all three volunteers taking part in the assay were collected to measure facemask contamination occurring due to splattering. All three volunteers then wore a new respirator and remained at their positions for 5 min, allowing any potential air droplets/aerosols to settle on the clean masks. This process was performed three times corresponding to three 5 min intervals: 0–5 min post-hand drying, 5–10 min post-hand drying, and 10–15 min post-hand drying (Figure 1B), to assess potential deposition of aerosolized particles. Data of all 24 assays were included in the study to represent the diversity of users of public washrooms.

### Bacteriophage recovery from masks

All N95 respirators were carefully bagged upon collection and transferred to the lab for testing. The mask outer layer was removed with the aid of sterile scissors, placed in a sterile petri dish and saturated with 2 ml of DNA/RNA shield solution (Zymo Research, Germany) (16). After 5 min, the mask outer layer was transferred to a 15 ml falcon using sterile forceps. The tube was centrifuged for 1 min at 3,300 g, the supernatant was recovered and stored at 4°C until DNA extraction.

### DNA extraction and viral quantitation

DNA extraction was performed from 400 μl of elute using the QIAamp 96 Virus QIAcube HT Kit (Qiagen, Germany). Bacteriophage quantification was performed using quantitative PCR (qPCR) targeting the P3 gene of bacteriophage PR772, as previously described (2). Briefly, qPCR reactions containing final concentration of SYBR Green 1x Master Mix (Qiagen, Germany), 0.6 μM of primers specific for gene P3 (primer forward: 5′-CCCATTAAGTACGGCGATGTTATG-3′; primer reverse: 5′-GGCAAGCGGAACCCAATAG-3′) (11) and 18.75 ng of DNA template were prepared to a final volume of 15 μl. Reactions were analyzed in a Rotor-gene Q (Qiagen, Germany) using the following conditions: 95°C for 5 min, and 95°C for 10 s, 58°C for 15 s, 72°C for 20 s, repeated for 45 cycles. Standard curves were included in triplicate on each qPCR run and used to convert threshold cycle values to copies per μL of template. Limit of detection was established at 500 copies. Each DNA extraction was analyzed in duplicate.

### Statistical analysis

SPSS version 23 was used for data analysis. Statistical significance was assessed using a two-sided Mann-Whitney U test for independent samples, i.e., to compare samples collected during jet air dryer vs. paper towel hand drying. The test used a 90% confidence interval with *p* < 0.1 considered statistically significant.

## Results

Overall, 24 hand drying assays were performed by 14 volunteers to determine the potential for microbial contamination of facemasks following hand drying using either paper towels or a jet air dryer.

### Contamination of N95 mask as result of splattering

**Figure 2:**
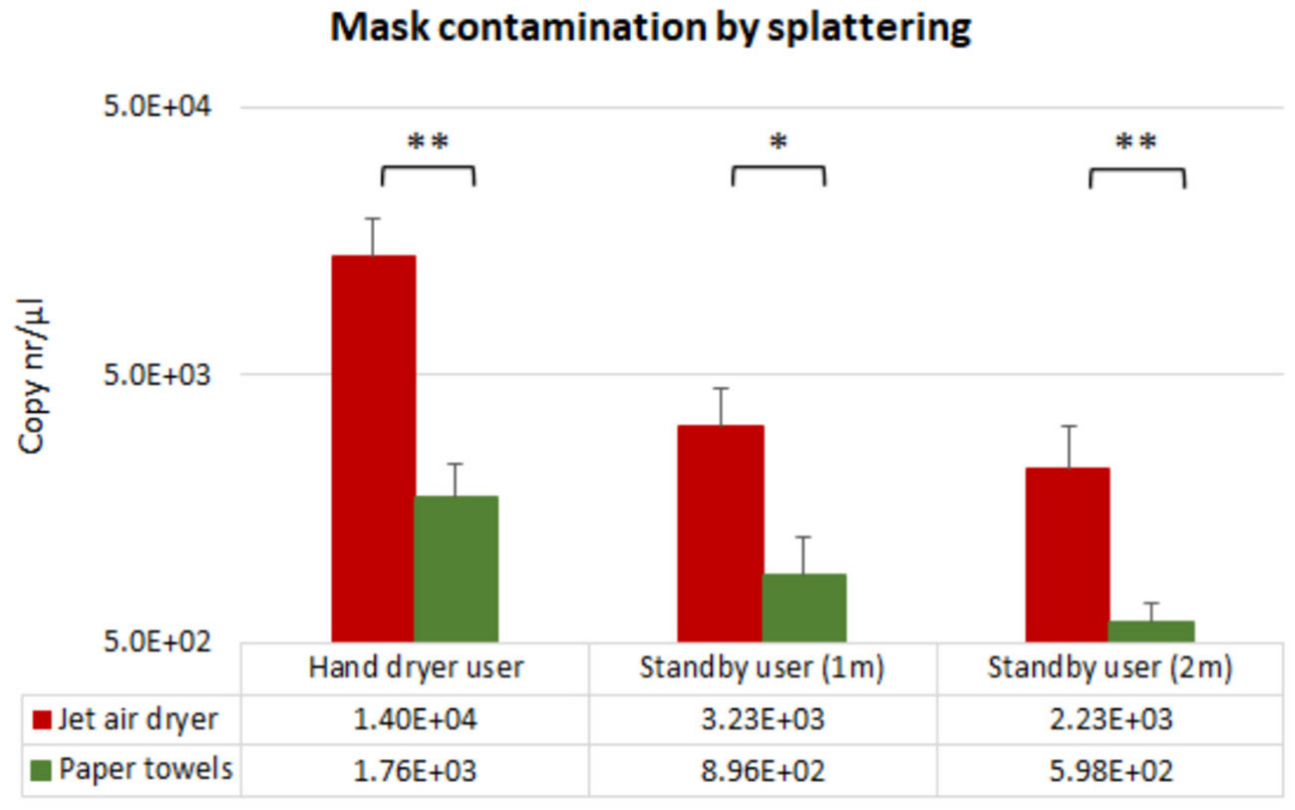
Mean qPCR results for PR772 bacteriophage recovered from N95 masks immediately following hand drying (splattering). Bacteriophage recovery is presented as logarithms of gene copy numbers to achieve normal distribution. Concentration of PR772 bacteriophage in the hand immersion solution was 6.5 x 10^8^ copies/μl. Single asterisk and double asterisk correspond to *p < 0.1 and **p < 0.05 significant difference between hand drying methods using the Mann-Whitney test, respectively.

Contamination as result of splattering was investigated by recovering N95 masks worn by the volunteers while performing the hand drying, and by the standby users stationed at 1 m and at 2 m distance of the hand drying unit while hand drying was performed. Bacteriophage splattering on the masks as result of hand drying was observed with both hand drying methods. Mask contamination of the volunteers' occurring while performing the hand drying was observed in 29% (=7/24) of the paper towels assays, while 88% (=21/24) of the jet air dryer tests were positive for bacteriophage recovery. Following hand immersion in a PR772 bacteriophage solution (6.5 x 10^8^ copies/μl), mask contamination of the users during hand drying was 10-fold higher when the jet air dryer was used (1.4 x 10^4^ copies/μl), compared with paper towels (1.76 x 10^3^ copies/μl) (Figure 2).

Similarly, mask contamination of participants standing at 1 and 2 m distance of the hand drying unit was also 10-fold higher at both distances (Figure 2), when using a jet air dryer. The percentage of positive assays for the standby user position was more than 20% higher in the jet air dryer assays (*n* = 7) compared with paper towel use (*n* = 2).

### Recovery of bacteriophage resulting of droplet deposition

Following hand drying, all participants remained at their defined positions, i.e., by the hand drying station, at 1 m, and at 2 m distance from the hand dryer area. By having the volunteers swap to a fresh N95 respirator after 5 and 10 min (over 15 min in total), we were able to assess if each hand drying method created aerosols/droplets dispersion in the room environment that could deposit on the masks surface when subjected to natural air displacement, including that associated with regular breathing.

**Figure 3:**
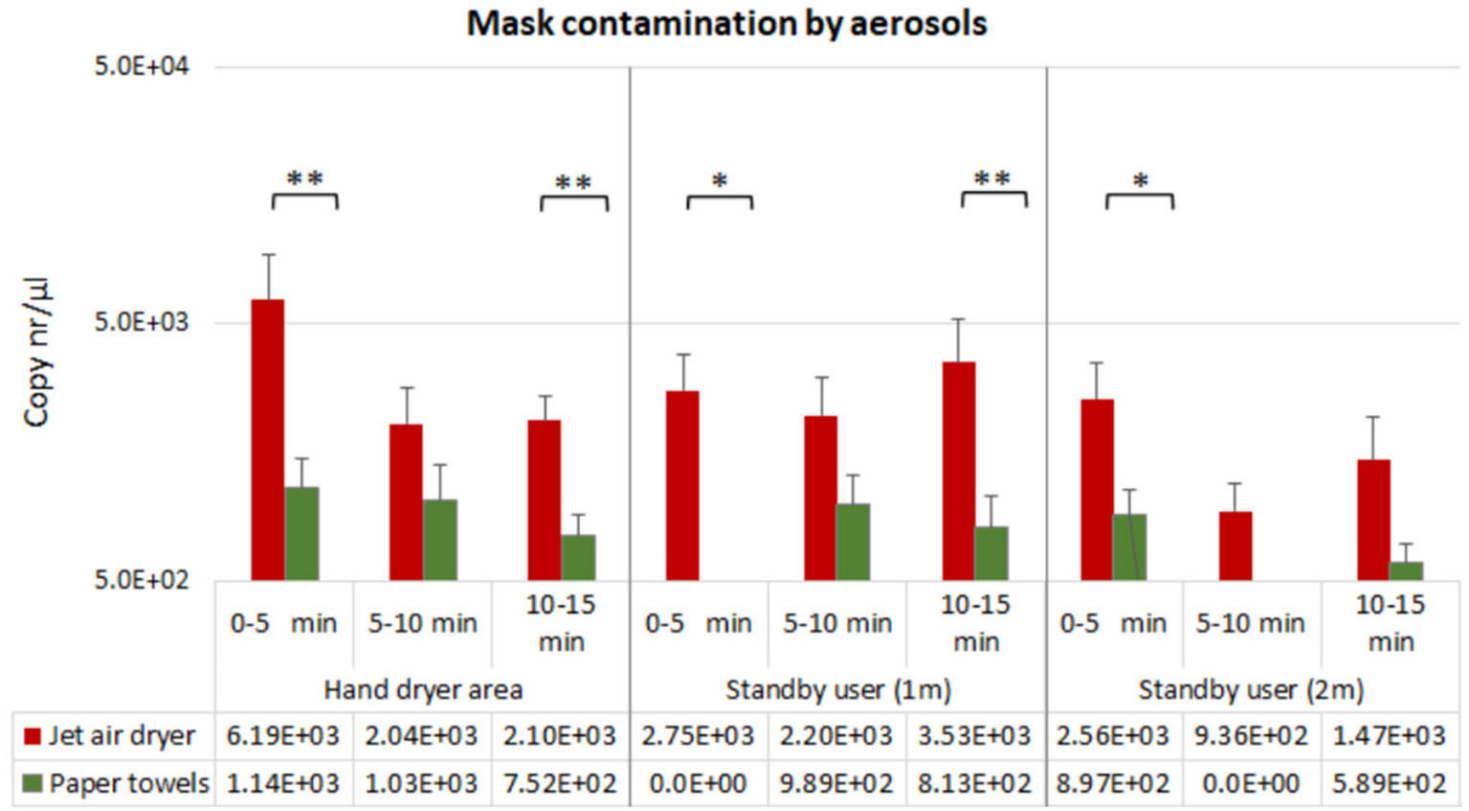
Mean qPCR results for PR772 bacteriophage recovered from N95 masks at each tested position at 5, 10, and 15 min post-hand drying. Bacteriophage recovery is presented as logarithms of gene copy numbers to achieve normal distribution. Single asterisk and double asterisk correspond to *p < 0.1 and **p < 0.05 significant difference between hand drying methods using the Mann-Whitney test, respectively.

Hand drying using jet air dryer resulted in mask contamination by droplets/aerosols in all positions and timepoints (*n* = 9), whereas this was observed in 78% of the testing points (*n* = 7) when paper towels were used (Figure 3).

For both hand drying methods, mask surface contamination by droplet deposition was higher in the first 5 min following hand drying, in all distances; however, bacteriophage contamination was significantly higher when the jet air dryer was used, compared with paper towel use (Figure 3).

When hand drying with paper towels, lower mask contamination levels were observed in the hand dryer area, suggesting lower aerosolization of viral particles (Figure 3). Furthermore, recovery of bacteriophage aerosolized particles in this area decreased across time when using paper towels, with less contamination observed at 5–10 min and 10–15 min intervals post-hand drying, compared with the 0–5 min interval. Dispersion of viral particles to 1 and 2 m from the hand drying station was inconsistently observed with paper towels. We found 4–17% (i.e., 1/24–4/24) of the assays performed were positive for bacteriophage droplets in N95 respirators at these distances, with the recovered viral loads being 10-fold lower compared with use of jet air dryer, in every position and timepoint (3/24–9/24 of assays showed bacteriophage contamination when using jet air dryer).

Interestingly, jet air dryer use resulted in increased mask surface contamination at 15 min post-hand drying, compared to the 5–10 min interval, for all distances investigated, suggesting aerosolization and dispersion of small particles, that can remain airborne for longer.

## Discussion

Hand drying is an essential step of hand hygiene and can effectively aid with microbial removal from poorly washed hands, preventing pathogen dissemination beyond the toilet (2, 17, 18). Paper towels have been shown more efficient in this process, alongside having a lower potential to disperse droplets (1, 3, 19–21) and lower particle aerosolization, compared with jet air dryers (1, 21, 22). However, it is unknown if the particles aerosolized during hand drying can also have an impact in microbial dissemination in the washroom environment and pose an infection risk. This question becomes particularly relevant when we consider airborne transmissible pathogens, such as SARS-CoV-2 or influenza, and whether aerosols formed during hand drying can constitute a pathogen transmission risk. Some evidence of SARS-CoV-2 transmission in public toilets has been found, with contaminated air and surfaces being considered the main potential cause (23). Viral particles expired by COVID-19 infected individuals, or aerosols resulting from feces or urine are known potential sources for air contamination (23, 24), but the risk posed by hand drying for the dissemination of respiratory virus remains unclear.

To investigate this, we used a bacteriophage as a surrogate (11) to represent microbial/viral contamination and assessed droplet and aerosol deposition on N95 respirators at different distances and time intervals after hand drying. Overall, higher viral contamination was observed at all positions and timepoints investigated when jet air dryer was used, compared with paper towels. This suggests greater production of aerosols during jet air dryer use, compared with paper towels, and larger heterogeneity of viral particles emitted during hand drying. Small particles would travel further and remain airborne for longer, explaining the increased viral recovery reported at 15 min and at 2 m distance from the hand drying station, when the jet air dryer was used.

The higher load of bacteriophage particles traveling to 1 m and 2 m distance observed when jet air dryer was used, agrees with previous reports of droplets dispersing up to 2.2 m when using this method of hand drying (3, 20, 21). Although mask contamination of standby users at 2 m was also observed when paper towels were used for hand drying, this was 10-fold lower compared with the jet air dryer. Overall, when using a jet air dryer, microbial contamination dispersed further in the washroom environment and settled on masks at different timepoints.

This pilot study had several limitations. Despite the use of standardized hand drying methods, some variability associated with different users is inherent, and it would be found in the regular users of a public washroom facility. The bacteriophage concentration used aimed to represent poorly washed/ unwashed/ contaminated hands, therefore a step of hand washing was not part of the study design and this may exaggerate the potential for airborne microbe transmission. Nonetheless, hands contaminated due to poor hand washing in public toilets remain common, with one study reporting over 83% of users not adequately washing their hands in the first weeks of COVID-19 pandemic (25), while other studies reported improvement of hand hygiene by healthcare professionals during the pandemic (26, 27), but a decline in compliance over time (27). The infectious dose of inhaled virus remains unclear and so it cannot be assumed, of course, that facemask contamination is synonymous with infection. Nevertheless, our results suggest that the two examined hand drying methods have different potential for airborne dispersal of microbes in public washrooms, and so differing levels of environmental contamination, risk of virus inhalation and infection. Future studies should include hand washing to determine how this step may impact aerosol dispersion.

## Conclusion

Our study investigated potential mask contamination associated to the hand drying method and determined that hand drying can cause aerosolization of microbial/viral particles, promote their spread to the washroom environment and contaminate other users up to a 15 min period post hand drying. The risk of facemask contamination was significantly increased when using a jet air dryer compared with paper towels. The use of facemasks has provided protection to the public and healthcare workers during the COVID-19 pandemic (5, 7, 9, 10, 28). As mandatory mask use has been largely lifted in western countries, users of public washroom will be more directly exposed to potentially contaminated aerosolized particles, by SARS-CoV-2 and other airborne transmissible pathogens such as influenza, rhinovirus or respiratory syncytial virus, the prevalence of which can be expected to increase following easing of COVID-19 restrictions (4). As such, it is important to consider the potential for virus dissemination during hand drying. Based on our observation, hand drying with paper towels is associated with a lower risk of droplet and aerosol dispersion compared with use of a jet air dryer.

## Data availability statement

The raw data supporting the conclusions of this article will be made available by the authors, without undue reservation.

## Ethics statement

The approval of bacteriophage use in volunteer studies was approved by the School of Medicine Research Ethics Committee, University of Leeds (reference MREC 18-094). Written informed consent for participation was not required for this study in accordance with the national legislation and the institutional requirements.

## Author contributions

IM was involved in methodology, formal analysis, data curation, and writing–original draft. KB was involved in investigation and writing–review and editing. MW was involved in study conceptualization and writing–review and editing. All authors have read and agree to the published version of the manuscript.

## Funding

This study was supported by the European Tissue Symposium. The funder had no involvement in project design, data collection, analysis or interpretation, or in the manuscript preparation.

## Conflict of interest

Author MW has received honoraria for consultancy work, financial support to attend meetings and research funding from Astellas, AstraZeneca, Abbott, Actelion, Alere, Bayer, bioMérieux, Cerexa, Cubist, Da Volterra, Durata, ETS, Merck, Nabriva Therapeutics plc, Pfizer, Qiagen, Roche, Seres Therapeutics Inc., Synthetic Biologics, Summit and The Medicines Company. The remaining authors declare that the research was conducted in the absence of any commercial or financial relationships that could be construed as a potential conflict of interest.

## Publisher's note

All claims expressed in this article are solely those of the authors and do not necessarily represent those of their affiliated organizations, or those of the publisher, the editors and the reviewers. Any product that may be evaluated in this article, or claim that may be made by its manufacturer, is not guaranteed or endorsed by the publisher.
