# Supplementary material for: Assessment of potential for viral contamination of user and environment via aerosols generated during hand drying: A pilot study
Source: Front Public Health. 2022 Oct 25;10:1010802. doi: 10.3389/fpubh.2022.1010802 (PMC9641239; doi:10.3389/fpubh.2022.1010802)
Supplement: Supplementary file 1 [file Image_1.pdf]

Supplementary Material

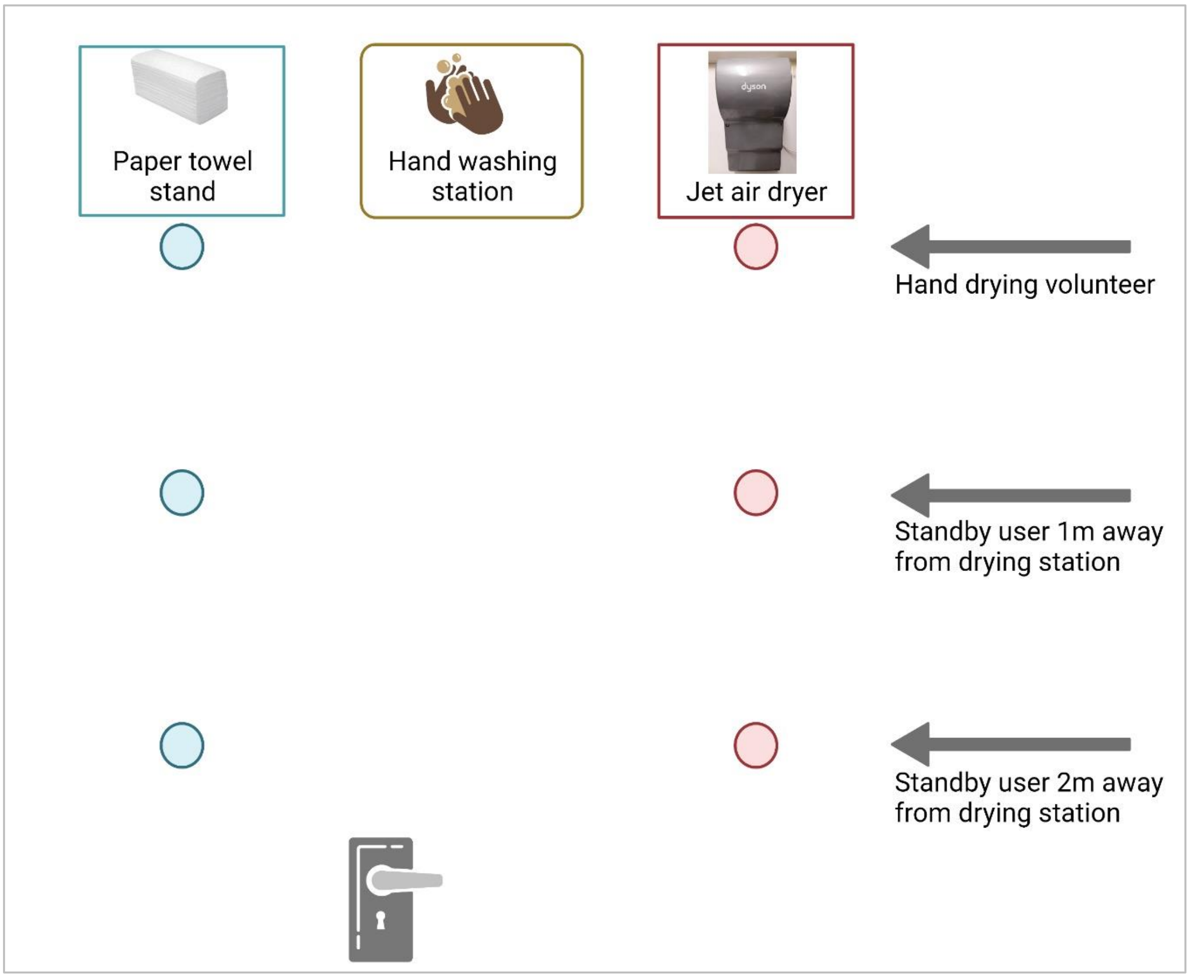

Supplementary Figure 1. Schematics of the room layout where hand drying experiments were performed. Figure created with Biorender.com.
